# Supplementary material for: Color-switching hydrogels as integrated microfluidic pressure sensors
Source: Sci Rep. 2024 Mar 15;14:6333. doi: 10.1038/s41598-024-56140-z (PMC11319356; doi:10.1038/s41598-024-56140-z)
Supplement: Supplementary file 1 — Supplementary Information 1. [file 41598_2024_56140_MOESM1_ESM.pdf]

# Supplementary material

# Color-switching hydrogels as integrated microfluidic pressure sensors

Lucie Ducloué[1], Md. Anamul Haque[2,4], Martyna Goral[1], Muhammad Ilyas[2], Jian Ping Gong[2,3], Anke Lindner[1]

[1] Laboratoire de Physique et Mécanique des Milieux Hétérogènes, UMR 7636, CNRS, ESPCI Paris, PSL Research University, Université Paris Cité, Sorbonne Université, Paris, FR-75005, France

[2] Laboratory of Soft & Wet Matter, Faculty of Advanced Life Science, Hokkaido University, Sapporo, Hokkaido 001-0021, Japan

[3]Institute for Chemical Reaction Design and Discovery (WPI-ICReDD), Hokkaido University, Sapporo 001-0021, Japan

[4]Department of Chemistry, University of Dhaka, Dhaka-1000, Bangladesh

## Color spectra of a free-standing gel

Color spectra are obtained using gel samples that have not yet been implemented into the microfluidic pressure sensor. Supplementary Fig. 1 (a) shows the experimental setup used to compress the gel, placed between a substrate and a glass slide. A well-controlled force is applied via a load cell and the displacement is measured with a displacement sensor. At the same time, the color spectrum is measured in reflection with a spectrometer as shown in Supplementary Fig. 1 (b) for a gel of thickness $1\,\mathrm{mm}$. Supplementary Fig. 1 (c) shows color pictures of the same gel disc under compression and the corresponding hue-value intensities. Fig. 1 (c) in the main text shows similar color images and hue intensities for a different gel of thickness $140\,\mu\mathrm{m}$.

## Hue value versus color spectra and Gel moduli

In Supplementary Fig. 2 (a) we represent the maximum reflected wavelength $\lambda_{max}$ measured from the color spectra (Supplementary Fig. 1 (b)) as a function of the applied pressure. The pressure is obtained from the applied force divided by the actual surface of the disc obtained from the images (Supplementary Fig. 1 (c)). A similar graph is shown in the main text Fig. 1 (d) for a different gel of thickness $140\,\mu\mathrm{m}$. The inset shows the hue value as a function of the maximum reflected wavelength. A saturation of the hue-value intensity for wavelengths below $480\,\mathrm{nm}$ is observed, in agreement with the inset of Fig. 1 (d). Supplementary Fig. 2 (b) shows the determination of the Young's modulus of two different gels from pressure-deformation (corresponding to stress-strain) curves. Data for the thick gel correspond to the experiment represented in Supplementary Fig. 1 and data for the thin gel to the experimental results presented in Fig. 1 (d). Typical moduli between $10\,\mathrm{kPa}$ and $100\,\mathrm{kPa}$ are obtained.

## Sensor calibration

The calibration of the microfluidic sensors is done independently from the characterization of the free-standing gel discs. In order to link the hue value to an applied pressure, each sensor disc has been directly calibrated. In particular, in Supplementary Fig. 2 (c) we plot the resulting hue-value measured from camera recording as a function of imposed pressure in the microfluidic channel for a sensor of $1\,\mathrm{mm}$ diameter from the set-up used for Fig. 2 (d). The maximum pressure applied in our experiments is $140\,\mathrm{mbar}$ which results in a range of measurable pressures for the smaller sensors from $60\,\mathrm{mbar}$ to $140\,\mathrm{mbar}$. Pressures below $60\,\mathrm{mbar}$ are measured with $4\,\mathrm{mm}$

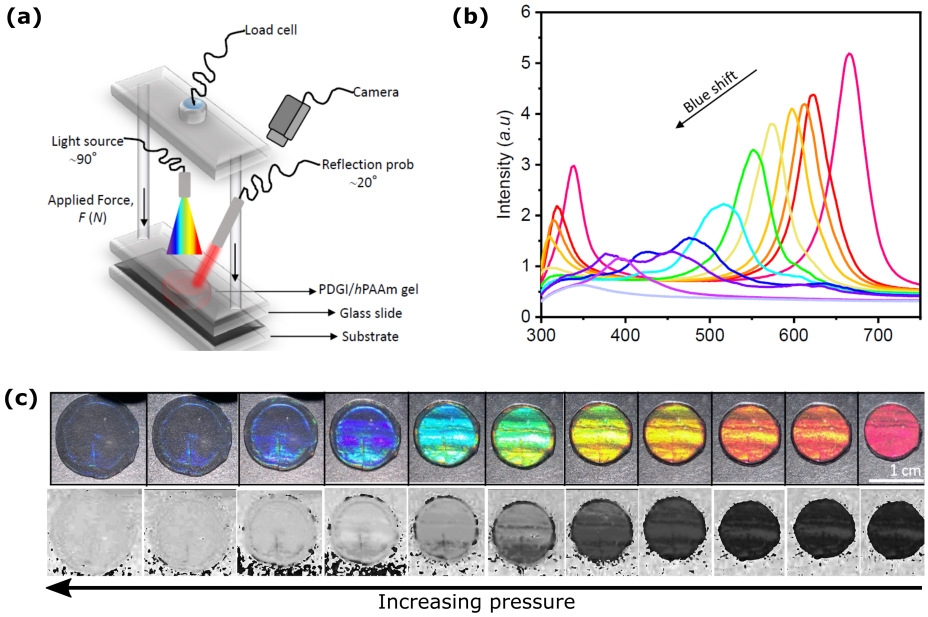


**Supplementary Figure 1.** (a) Scheme of the experimental set-up measuring the reflected wavelength of a free-standing 1 mm thick gel disc under applied pressure, using a spectrometer. (b) Wavelength intensity distribution for several imposed pressures, for a free-standing gel disc. $\lambda_{max}$ corresponds to the wavelength at maximum intensity. (c) Corresponding photographs and hue intensity pictures.

diameter sensors (Figure 1 (g)). The calibration for several sensors of diameter $140\,\mu\text{m}$ is presented in Fig. 1 (g) in the main text.

## Membrane-gel coupling theory and model

Using a thin-plate approximation for the membrane, the coupled response of the membrane and the gel under pressure can be modeled as a bending plate resting on an elastic foundation[1], described by the following equation.

$$\left(\frac{d^2}{dr^2}+\frac{1}{r}\frac{d}{dr}\right)\left(\frac{d^2w}{dr^2}+\frac{1}{r}\frac{dw}{dr}\right)=\frac{q-kw}{D} \tag{1}$$

Where $r$ is the radial position, $w$ the membrane deflection, $q$ the uniform load or pressure, $k$ represents the gel reaction defined here as the ratio of the gel Young's modulus to its thickness $k=E_g/H_g$, and $D$ is the bending modulus of the PDMS membrane. In the Winkler foundation model, this problem can be solved analytically[2].
Here we imposed the membrane Young's modulus $E_m = 1\times10^6$ Pa, thickness $H_m = 100\,\mu\text{m}$, Poisson's ratio $\nu = 0.5$ and bending modulus $D=\frac{E_m H_m^3}{12(1-\nu^2)}$, as well as typical values for the gel Young's modulus $E_g = 3\times10^4$ Pa and thickness $H_g = 170\,\mu\text{m}$. The membrane is clamped at the circular cavity edges.
First we fix the sensor radius to $R=2\,\text{mm}$ and vary the pressure load $q$ from $0\,\text{mbar}$ to $120\,\text{mbar}$ with an increment of $5\,\text{mbar}$. This solution gives a deformation profile (represented in the Supplementary Fig. 3 (a)) which is in good qualitative agreement with the measured profiles: the obtained deformation shape matches the measured hue-value profiles across the sensor diameter, visible in the the main article Fig. 1(f). In particular, the small dip in deformation in the middle of the sensor at high pressure is reproduced by the simulations. Using this simple linear model, we do not expect full quantitative agreement, in particular at large deformation and high pressures. However, the observed order of magnitude of deformation agrees between experiments and modeling and is in both cases observed to be around 30% at maximum deformation.

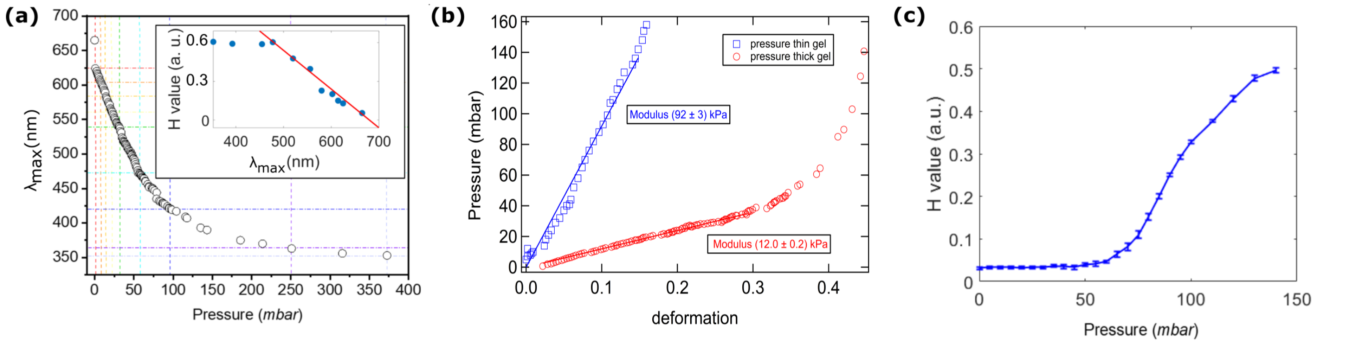


**Supplementary Figure 2.** (a) Maximum reflected wavelength as a function of pressure measured by spectrometer for a free-standing 1 mm thick gel disc and associated Hue-value (inset) from the same experiment as shown in Fig.2. The line is a guide for the eye. (b) Pressure as a function of gel deformation, for both thin and thick gel discs of respective thicknesses 140 μm and 1 mm and corresponding Young's moduli. (c) Calibration curve for a sensor of 1 mm diameter.

This linear modeling also gives insight into the size-dependence of the response of the sensors shown in the Supplementary Fig. 3 (b), for a fixed pressure $p = 25\,\text{mbar}$ and varying the radius from $100\,\mu\text{m}$ to $2\,\text{mm}$. The deflection gets smaller with decreasing radius, thus requiring more pressure to deform the membrane. This clearly shows that higher pressures can be measured by decreasing $R$. It is essentially due to the bending response of the membrane, which by integration scales with $R^4$. We make use of this scaling in the design of our sensors and design two different sizes: large sensors sensitive to small pressures (working range 20-80 mbar) and small sensors sensitive to larger pressure (working range 60-150 mbar). The coupling of the gel response with the membrane thus greatly extends the working range of a given hydrogel sample towards larger pressures.

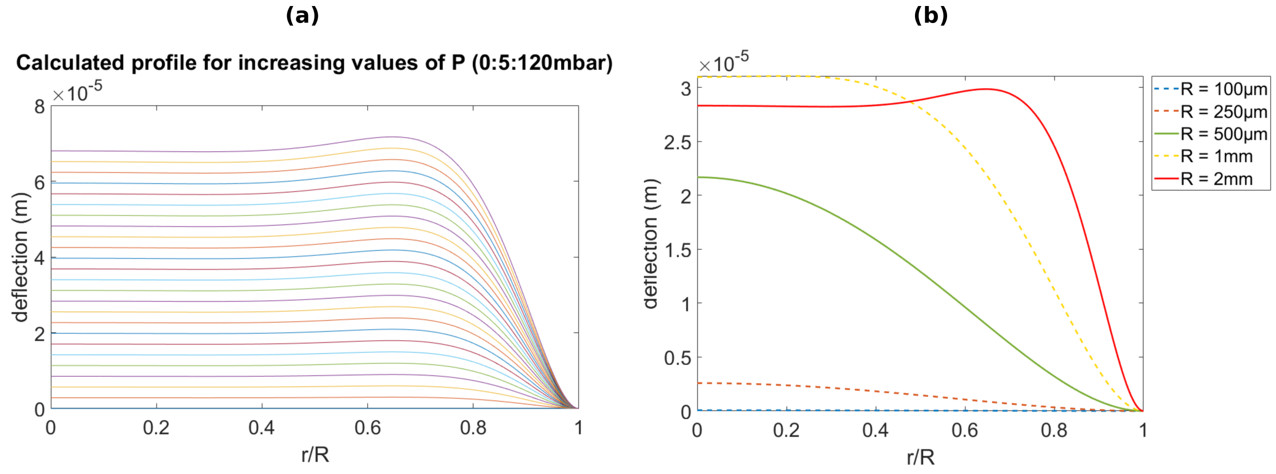


**Supplementary Figure 3.** Analytical results of the membrane-gel coupling model : (a) deflection of the membrane with increasing pressure from 0 mbar to 120 mbar with an increment of 5 mbar, for a radius of 2 mm. (b) Deflection of the membrane for several radii, at a fixed pressure of 25 mbar.

## Dynamic response

The dynamic response of the device under flow was tested by imposing an oscillating pressure at the channel inlet for the chip shown in the main article Fig. 2. This profile is shown in Supplementary Fig. 4 as a blue dashed curve: a steady pressure is imposed for a few seconds, then the pressure oscillates with a period of 5 s with a mean value

equal to the previously applied steady pressure. After 3 periods, it is set to 0. All sensors exhibit an oscillating response. The response of the first large sensor (closer to the inlet) is plotted in Supplementary Fig. 4 (red curve). The response time of the whole chip has been estimated by fitting an exponential to the measured pressure increase after the step increase of the applied pressure and is quantified as the response time of the whole chip to be around $1.3\,\mathrm{s}$. The period of oscillations is conserved with a phase shift of comparable order of magnitude of about $1\,\mathrm{s}$ measured by taking the average of the time shifts between the maxima and minima of both curves. The amplitude of the measured oscillating pressure is decreased compared to the applied pressure because of the large response time of the system compared with the period. The mean value observed is smaller compared to the applied pressure due to the pressure drop upstream of the sensor. The results show no visible hysteresis in response of the sensor.

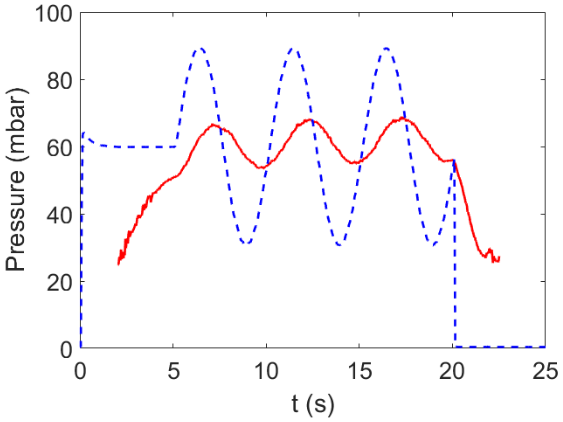


**Supplementary Figure 4. Dynamic response of the sensors**: an oscillating pressure is imposed upstream of the inlet (blue dashed curve); the response of the first actuated 4 mm sensor is shown in red (the sensor is only activated for pressures above 20 mbar): the period is the same, there is no up/down asymmetry, and the mid-value is shifted towards a lower pressure because of the pressure drop upstream of the sensor; there is also a shift and the sensed amplitude is smaller, due to the characteristic response time of the whole chip.

## References

**1.** Timoshenko, S. & Woinowsky-Krieger, S. *Theory of Plates and Shells*. Engineering mechanics series (McGraw-Hill, 1959). URL `https://books.google.ca/books?id=rTQFAAAAMAAJ`.

**2.** Al-Hosani, K. I. A. *Stress analysis of thin and thick plates on elastic foundations using boundary and finite element methods*. Ph.D. thesis (1991).
